# Health behaviours and well-being among older adults with a Surinamese migration background in the Netherlands

**DOI:** 10.1186/s12889-022-14414-z

**Published:** 2022-11-02

**Authors:** Warsha Jagroep, Jane M. Cramm, Semiha Denktaş, Anna P. Nieboer

**Affiliations:** 1 Department of Socio-Medical Sciences, Erasmus School of Health Policy & Management, Erasmus University Rotterdam, Rotterdam, the Netherlands; 2 Department of Psychology, Education and Child Studies, Erasmus School of Social and Behavioural Sciences, Erasmus University Rotterdam, Rotterdam, the Netherlands

**Keywords:** Diet, Health behaviour, Older surinamese adults, Physical activity, Smoking, Social activity, The Netherlands, Well-being

## Abstract

**Background:**

This study aims to identify the relationships between health behaviours (healthy diet, physical activity, not smoking and social activity) and well-being among older adults with a Surinamese background.

**Methods:**

Community-dwelling older adults (≥ 70 years) with a Surinamese background living in Rotterdam, the Netherlands, were identified by the municipal register. A survey study was conducted to assess background information, health behaviours (healthy diet, physical activity, not smoking and social activity) and well-being. Multiple regression analyses were performed to assess the relationships of health behaviours with well-being while controlling for background characteristics.

**Results:**

Average age of participants was 76.2 (4.9) years, slightly more than half of them were female (54.2%). Almost half of the participants had a low-income level (49.6%). More than half of the participants met the Dutch guidelines of fruit intake (63.0%) and vegetable intake (62.8%). Less than half of the participants met the guidelines of fish intake (40.9%) and physical activity (39.8%). The majority of the participants were non-smokers (87.9%). Most of the participants had daily contact with family/friends (90.9%) and slightly more than half of the participants visited family/friends often (53.6%). Looking at the health behaviours, a positive relationship was found between eating enough fruit (*β* = .109; *p* ≤ 0.05) and vegetables (*β* = .135; *p* ≤ 0.01), physical activity (*β* = .164; *p* ≤ 0.001) and often visiting family/friends (*β* = .158; *p* ≤ 0.001) with well-being.

**Conclusion:**

This study suggests that next to traditional health behaviours also social activity is an essential health behaviour for the well-being of older Surinamese adults. Research about health promotion should expand its focus by including social activity as health behaviour.

**Supplementary Information:**

The online version contains supplementary material available at 10.1186/s12889-022-14414-z.

## Introduction

Surinamese people are one of the largest communities with non-Western migratory backgrounds in the Netherlands. The number of older Surinamese adults (≥ 55 years) in the Netherlands has more than quadrupled between 1990 and 2020 [1]. Surinamese people migrated to the Netherlands from Surinam, a former Dutch colony in South America. Surinam's population is diverse, with Surinamese Chinese, Surinamese Creole, Surinamese Javanese and Surinamese Hindustani, all having different cultures and geographical origins. In the Netherlands, the main subgroups are Surinamese Creole (West African descent) and Surinamese Hindustani (Indian descent).

A recent literature review on health and well-being among older migrants (including Surinamese older adults) in the Netherlands, indicated that research into well-being of older migrants in the Netherlands is scarce; none of the included studies involved the well-being of Surinamese older adults (only Turkish and Moroccan older adults) [2]. However, studies about aspects related to well-being, such as loneliness, were discussed, indicating that Surinamese older adults are more often lonely due to disadvantaged health, socio-economic status and low social participation compared to their native counterparts.

Maintaining a healthy lifestyle such as regular engagement in physical activity (PA), eating healthy and retaining from smoking is well known to be beneficial to peoples’ well-being [3–5]. Furthermore, findings from earlier studies show that people who engage in fewer health risk behaviours are more likely to be satisfied with their lives [6]. Besides traditional health behaviours such as PA, a healthy diet and not smoking, also older adults’ ability to stay socially active and connected to others seems to be critical to sustaining their well-being [7].

In general, in all ages health behaviours are known to differ between immigrants and natives [8–10], which may be influenced by certain social and cultural beliefs and/or values [11, 12]. According to previous research, beliefs about food vary widely from country to country, which can be influenced by social customs, religion and shared cultural values [13, 14]. Many people with a migration background continue to eat foods from their country of origin, in addition to foods from the host country [15]. Also, among the Surinamese population in the Netherlands, research shows that traditional Surinamese dishes and vegetables play an important role in the dietary behaviour of Surinamese people, especially among the first generation [16–18]. The cooking and eating practices of Surinamese people are deeply rooted in cultural beliefs and values (e.g. bitter vegetables are good for health) [19]. In addition, Surinamese older adults have indicated that the available options in the Netherlands regarding physical activities are insufficiently adapted to their cultural habits, such as inexperience with recommended ‘Dutch’ activities such as cycling and unavailability of programs which they prefer (e.g. dancing to Surinamese music), which discourages them to engage in a physically active lifestyle [20]. Furthermore, in Surinamese culture being curvy is often regarded as a sign of beauty, prosperity and strength in their community; this norm is likely to discourage them from being physically active [20]. Religion and culture play an essential role in shaping smoking behaviours, among Surinamese people social norms discourage women to smoke which does not apply for men [21, 22]. Older adults with a Surinamese background are mainly socially active within their own social network (family and friends). Social activities outside the household (e.g. theatre, going to a restaurant) and social clubs (e.g. sport, music) are less popular within this population [23].

In the Netherlands, people with a Surinamese background are more likely to have (multiple) chronic diseases (e.g., type II diabetes mellitus) [24, 25]. Research indicates that chronic diseases are associated with impaired well-being [26]. Engaging a healthy lifestyle plays an essential role in the prevention of many chronic diseases, such as type II diabetes mellitus and might eventually have a positive impact on sustaining well-being [27].

While previous research has indicated that a healthy lifestyle is associated with well-being among the general older population, there is still a lack of studies investigating the relationship between a healthy lifestyle and well-being among the Surinamese population in the Netherlands. The present study aims to examine the relationship between health behaviours (healthy diet, physical activity, not smoking, and social activity) and well-being among older adults with a Surinamese migration background in Rotterdam, the Netherlands.

## Methods

### Population

Surinam is a former Dutch colony in South America that gained independence in 1975. People from Surinam have migrated to the Netherlands mainly because of the unstable political situation in Surinam and to seek higher education and work [28]. As Dutch is an official language of Surinam and is used in education, government, and the media, most Surinamese people speak it well. The health of older Surinamese adults in the Netherlands is worse than that of the native Dutch older population. Older Surinamese adults are more likely to develop (multiple) chronic diseases (e.g., type II diabetes mellitus) and to experience mental health problems than are their native counterparts [24, 25, 29–31]. Additionally, older Surinamese adults have a greater risk of mortality from these chronic diseases than do their native counterparts with the same socioeconomic backgrounds or education levels [24, 29, 30, 32].

### Recruitment and data collection

Community-dwelling older adults (≥ 70 years) with a Surinamese background living in Rotterdam, the Netherlands, were identified by the municipal register. Participants were asked to participate in the study between March 2020 and June 2020. A written questionnaire was sent to participants by post with a self-addressed envelope, followed by a postal reminder. The aim of the study and its anonymous and voluntary nature was explained to participants by an information leaflet. The first authors’ contact details were provided to participants in case they had additional questions. In total 2749 participants were approached. Thirty-four participants were excluded, as they resided in nursing homes, due to serious medical issues (e.g., dementia, revalidation), change of address or death. Of the remaining 2715 participants, 679 returned filled-in questionnaires (25% response rate), nested in 56 neighbourhoods.

### Measures

#### Well-being

The short version of the validated social production function instrument for the level of well-being (SPF-ILs) was used to assess well-being [33]. The overall well-being was assessed by measuring levels of social well-being (affection, behavioural confirmation, and status), physical well-being (comfort and stimulation), and overall well-being [33–36]. Examples of questions assessing social well-being are: ‘Do people really love you?’ (affection), ‘Do others appreciate your role in the group?’ (behavioural confirmation) and ‘Do people think you do better than others (status)’. ‘In the past few months, have you felt relaxed?’ (comfort) and ‘Are your activities challenging to you?’ (stimulation) are questions which assessed physical well-being. On a four-point scale, responses ranged from never (1) to always (4). The mean of the five subscales was used to calculate overall well-being. In this study, Cronbach’s alpha value for overall well-being was 0.85, indicating high internal consistency.

#### Dietary behaviour

Diet was assessed by evaluating participants’ fish, fruit, and vegetable consumption as an indicator of healthy eating. Guidelines of the Dutch Health Council regarding healthy eating were followed to distinguish between healthy and unhealthy diets [37]. Questions solicited about food quantity and frequency; we gave Surinamese examples in the questionnaire. We used the threshold value of two times a week *fish* consumption to distinguish healthy from unhealthy diets. Fish consumption was dichotomized into 0 (less than 2 times a week fish consumption) and 1 (≥ 2 times a week fish consumption). Participants were asked about their *fruit* consumption, a threshold value of two pieces of fruit every day of which half (one piece) can be replaced by one glass of fruit juice was considered to be healthy. Fruit consumption was dichotomized into 0 (fewer than two pieces of fruit every day) and 1 (≥ two pieces of fruit every day). Vegetable intake was assessed by asking participants whether they consumed 200 g of vegetables per day. Vegetable consumption was dichotomized into 0 (fewer than 200 g of vegetables per day) and 1 (≥ 200 g of vegetables per day).

#### Physical activity

Participants were asked to report how many days per week they were physically active (i.e., sports activities, exercise, house cleaning, work in the garden) for at least 30 min each day. This question comes from the validated and reliable short questionnaire to assess health-enhancing physical activity (SQUASH) [38, 39]. In the Netherlands, government agencies use this instrument to monitor the PA of the population [40]. Scores ranged from 0 (not being physically active at all for at least 30 min a week) to 7 (being physically active for at least 30 min every day of the week). The Dutch Standard for Healthy Physical Activity (≥ 5 days per week at least 30 min physically active) was used to dichotomize the PA scale into 0 (not meeting the standard) and 1 (meeting the standard of PA and being active for at least 30 min a day for at least five times per week) [41].

#### Smoking

Smoking was assessed by asking participants whether they currently smoked (0 = yes/ 1 = no).

#### Social activity

Social activity was assessed by asking participants how often they visited family and friends (never, once a year, several times a year, 1 – 3 times a month, once a week, several times a week). This variable was dichotomized into 0 (≤ 1 – 3 times a month visiting family or friends) and 1 (≥ 1 – 3 times a month visiting family or friends). Additionally, participants were asked how many people visited or called them per day (none, 1 – 2, 3 – 4, 5 – 6, 7 – 10 or > 10). This variable was dichotomized into 0 (no daily contact with family or friends) and 1 (daily contact with family or friends).

#### Multimorbidity

Multimorbidity was defined as having two or more chronic diseases [42]. The presence of chronic diseases was determined using a questionnaire that asked, "Have you had any of the following diseases or conditions in the preceding 12 months?" (0 = no, 1 = yes). A list of 10 chronic conditions (i.e. cardiovascular diseases, diabetes, lung diseases) developed by O’Halloran et al. [43] was provided. An option to report unlisted conditions was provided to participants, which resulted in a list of 51 additional conditions (e.g., limited vision and kidney failure). Most participants had osteoarthritis (*n* = 288) or diabetes (*n* = 249). These conditions were taken into account when we counted chronic diseases. Simple count was used in the analyses.

#### Socio-demographic variables

The questionnaire additionally asked participants for information on their age, gender (male or female), marital status (living alone/widowed/divorced or married/living with a partner), education and income.

Participants were asked to report their highest educational level completed in the Netherlands or abroad, with the option to write another response for unlisted forms of schooling. This variable was dichotomized into low education (completion of elementary school or less) and high education (more than elementary school).

Participants’ monthly household income, including social benefits, pensions and alimony was asked to determine their income level. Response options ranged from 1 (less than €1000 a month net) to 4 (€3050 or more a month net). An option was given with ‘Do not know/ do not want to tell’ as the fifth category. This variable was dichotomized into low income (less than €1350 a month net) and high income (€1350 or more a month net).

### Data analysis

In this study, SPSS software version 27 (IBM Corporation, Armonk, NY, USA) was used to analyse the data. Descriptive statistics were calculated to characterize participants’ health behaviours. Bivariate associations of variables expressing background characteristics, health behaviours (diet, PA, smoking and social activity) and well-being were examined. The assessment of multicollinearity yielded tolerance values > 0.3 and variance inflation factors < 3, indicating no sign of multicollinearity. The data met the assumptions of independent errors (Durbin-Watson value = 1.904) and normality of distribution. A histogram and a normal P-P plot of the standardized residuals indicated that the data contained approximately normally distributed errors. Multiple regression analyses were performed to assess the relationships of health behaviours with well-being while controlling for background characteristics. We tested whether neighbourhood level (level 2) significantly affected well-being by comparing -2 log likelihoods of the regression models containing well-being only and containing well-being and the neighbourhood level. The results showed that the neighbourhood level did not significantly affect well-being (889.613 vs. 886.624; *p* = 0.08). Listwise deletion of missing cases was used in the multivariate analyses (*n* = 413). Results were considered statistically significant when two-sided *p* values were ≤ 0.05.

## Results

Table 1 displays descriptive statistics for the older Surinamese adults. The average age of the 697 participants was 76.2 ± 4.9 (range 70 – 100) years and 54.2% of them were female. The majority of the participants reported being unpartnered (67.4%). Almost 40% of the participants reported low education and almost half of the participants reported having a low income. The mean number of multimorbidity was 1.6 ± 1.4 (range 0–8). Regarding a healthy diet, 40.9% of the participants met the standard of fish consumption. More than half of the participants met the standard of fruit (63.0%) and vegetable (62.8%) consumption. Nearly 40% of the participant met the standard of PA. The majority of the participants reported being non-smokers (87.9%). The majority of participants indicated having daily contact with family/friends (90.9%). Slightly more than half of the participants reported that they did visit their family/friends often (53.6%). Mean scores for overall well-being were 2.86 ± 1.39 (range 1 – 4).

**Table 1:** Descriptive statistics of the study population (*n* = 697)

Characteristic	*n*	Range	% or mean (SD)
Age	697	70 – 100	76.2 (4.9)
Gender (female)	697		54.2
Marital status (unpartnered)	680		67.4
Education (low)^a^	676		38.5
Income (low)^b^	654		49.6
Multimorbidity	681		1.6 (1.4)
Diet			
- Fish (meets standard)	685		40.9
- Fruit (meets standard)	605		63.0
- Vegetable (meets standard)	689		62.8
Standard physical activity (meets standard)	633		39.8
Smoking (no)	684		87.9
Social activity			
- Daily contact family/friends (yes)	662		90.9
- Visiting family/friends (often)	658		53.6
Well-being	669	1 – 4	2.86 (0.47)

^a^Low education is completion of elementary school or less

^b^Low income is less than €1350 a month net, *SD* Standard deviation

Table 2 shows the correlation between health behaviours (diet, PA, smoking and social activity) and well-being. Significant positive correlations were found between fruit consumption (*r* = 0.149; *p* ≤ 0.001), vegetable consumption (*r* = 0.247; *p* ≤ 0.001) and meeting the PA standard (*r* = 0.220; *p* ≤ 0.001) with well-being. Regarding social activity, a significant positive association was found between daily contact with family/friends (*r* = 0.097; *p* ≤ 0.05) and often visiting family/friends (*r* = 0.228; *p* ≤ 0.001) with well-being. No significant associations were found between fish consumption, smoking and well-being.

**Table 2:** Spearman correlations health behaviours (diet, physical activity, smoking and social activity) and well-being among older Surinamese adults

	Overall Well-being		
	*r*	*p*	95% CI
Age	-.082	.096	-.180 to .018
Gender (female)^a^	-.030	.547	-.129 to .070
Marital status (unpartnered)^b^	-.081	.101	-.179 to .019
Education (low)^c^	-.180	< .001	-.274 to -.082
Income (low)^d^	-.079	.109	-.177 to .021
Multimorbidity	-.171	< .001	-.266 to -.073
Diet			
- Fish (meets standard)^e^	-.015	.767	-.114 to .085
- Fruit (meets standard)^f^	.177	< .001	.079 to .272
- Vegetables (meets standard)^g^	.234	< .001	.138 to .326
Standard physical activity (meets standard)^h^	.238	< .001	.143 to .330
Smoking (no)^i^	-.007	.893	-.106 to .093
Social activity			
- Daily contact family/friends (yes)^j^	.069	.164	-.031 to .167
- Visiting family/friends (often)^k^	.210	< .001	.113 to .303

*r* correlation coefficient, *CI* Confidence interval,

^a^Reference category is male

^b^Reference category is partner

^c^Reference category is high education

^d^Reference category is high income

^e^Reference category is doet not meet fish consumption standard

^f^Reference category is does not meet fruit consumption standard

^g^Reference category is does not meet vegetable consumption standard

^h^Reference category is doest not meet physical activity standard

^i^Reference category is smoking

^j^Reference category is no daily contact with family/friends

^k^Reference category is seldom visits for family/friends

Table 3 demonstrates the association of health behaviours and well-being assessed by multiple regression analyses. After controlling for age, sex, marital status, education, income and multimorbidity, fruit consumption (*β* = 0.109; *p* ≤ 0.05) and vegetable consumption (*β* = 0.135; *p* ≤ 0.01) were associated well-being among older Surinamese adults. In addition, meeting the PA standard was associated with well-being (*β* = 0.164; *p* ≤ 0.001). Finally, often visiting family/friends was associated with well-being (*β* = 0.158; *p* ≤ 0.001) among older Surinamese adults.

**Table 3:** Relationships between health behaviours (diet, physical activity, smoking and social activity) and well-being, while controlling for socio-demographic characteristics, among older surinamese adults

	Overall well-being		
	Β	95% CI	*p*
Constant	2.722	2.112 to 3.435	.000
Age	-.001	-.009 to .008	.862
Gender (female)	-.047	-.135 to .041	.296
Marital status (unpartnered)	-.040	-.135 to .054	.401
Education (low)	-.122	-.207 to -.038	.005
Income (low)	.004	-.084 to .092	.925
Multimorbidity	-.111	-.192 to -.030	.008
Diet			
- Fish (meets standard)	-.021	-.102 to .061	.616
- Fruit (meets standard)	.109	.020 to .197	.017
- Vegetables (meets standard)	.135	.047 to .222	.003
Standard physical activity (meets standard)	.164	.081 to .248	.000
Smoking (no)	-.052	-.179 to .075	.423
Social activity			
- Daily contact family/friends (yes)	.065	-.088 to .219	.402
- Visiting family/friends (often)	.158	.077 to .239	.000

*B* Unstandardized regression coefficient, *CI* Confidence interval

## Discussion

This study aimed to investigate the relationship between health behaviours (diet, PA, smoking, and social activity) and well-being among older Surinamese adults in Rotterdam. the Netherlands. After controlling for background characteristics and multimorbidity, fruit and vegetable consumption, meeting the PA standard and social activity, specifically visiting family/friends, were associated with better well-being. Although similar findings have been obtained among other population groups [3–5, 7], this study is the first to show associations between multiple health behaviours and well-being among older Surinamese adults.

This study shows that *fruit and vegetable* consumption among older Surinamese adults is associated with better well-being. Several mechanisms may underlie the relationship between fruit and vegetable intake and well-being. For example, fruits and vegetables are rich in micronutrients such as vitamin C, which act as cofactors for neurotransmitters involved in positive motivational states [44].

A few studies have investigated diet among the Surinamese population in the Netherlands, however these studies have examined the relationship of diet with health related outcomes such as diabetes type II rather than well-being related outcomes [45]. Dietary interventions seem to have the potential to improve the diet quality of older adults [46]. In the Netherlands, dietary interventions for older adults are mainly focused at malnutrition and its health consequences such as decrease in muscle mass and decrease of the immune system [47]. Well-being measures are often omitted, despite that well-being improvements are promising to decrease health care utilization and expenditures [48]. In addition, these diet interventions are focused on the general older population, despite research suggests that culturally adapted interventions (e.g. use of traditional vegetables and species) might be more effective to promote a healthy diet [49]. Future dietary intervention should also focus on outcome measures related to well-being and involve the culture of the targeted population. This will help to develop effective dietary interventions, which will be beneficial for the health and well-being of older Surinamese adults (and the general (older) population) and potentially reducing health care expenditures.

A positive relationship of meeting *PA* guidelines with better well-being was seen in our study. PA releases endorphins in the body, which increase mood and energy, promoting well-being [50]. In the Netherlands, PA interventions are mainly focused to prevent and maintain diseases and limitations [51]; well-being outcome measures are also omitted during evaluation of PA interventions. Less than half of participants in our study met the PA guidelines of ≥ 5 days per week at least 30 min physically active. A recent study among older Surinamese adults showed that neighbourhood characteristics have an important role in supporting older Surinamese adults in engaging an active lifestyle [52]. Thus, policy makers should promote neighbourhood interventions that promote/support PA among the older Surinamese population, which might also be beneficial for the general population.

In our study, *visiting family/friends* was associated with significantly better well-being. Having *daily contact with family/friends* was not significantly associated with well-being in the multiple regression analyses. This indicates that digital social interactions do not replace face-to-face interactions. Therefore, it can be assumed that face-to-face social contact are more valuable compared to digital social interactions for the well-being of older Surinamese adults. In times of the COVID-19 pandemic, digital solutions were often used to maintain social contact with older adults due to social distancing. While digital solutions might be beneficial to maintain social distancing, this study shows that it is at the expense of the well-being of older Surinamese adults. Relevant example is, the relaxation of the COVID-19 measures by the Dutch government at the insistence of the ANBO (*Algemene Nederlandse Bond voor Ouderen*; General Dutch Association for Older Adults), since older adults became socially isolated [53]. Community-dwelling older adults were allowed to see one or two permanent persons physically, as long as, the COVID measures were maintained. Policy makers and future well-being interventions should consider the importance of face-to-face contact upon policy and intervention development. Visiting family and friends may be a way for older adults to receive social support, which may promote well-being through enhanced self-esteem [54]. This increased self-esteem, in turn, may promote optimism, positive affect, and better well-being [55]. Additionally, family members and friends may affect health-related behaviour [56]; social interaction and integration encourage the exchange of health information and persuasion and support, which may influence people, for example, to modify dietary and physical activity patterns [57].

Although Schiepers et al. [58], have indicated a positive relationship between greater *fish consumption* and well-being among a generally healthy population in the Netherlands, our study does not show this relationship. A possible explanation for this might be that our study was conducted among the older population. Indeed, an earlier study conducted among older men also showed no positive association between fish consumption and well-being [59].

In the present study, we found no relationship between *not smoking* and well-being. which is contrary to the findings of Lang et al. [60] who found a positive relationship. Daily exposure to stressors plays an essential role in smoking initiation and continuation [61]. People often attempt to ameliorate stress by smoking which gives them a temporary relief [62]. It might be that participants used smoking as a coping strategy for stress. Another possible explanation for this might be the low number of participants who smoked.

The Dutch government supports municipal health promotion, which focuses on changing people’s behaviour to promote health and/or prevent disease (https://www.loketgezondleven.nl/gezondheidsthema/stimuleren-gezond-gedrag). For older adults, the focus is on “healthy and vital aging.” Municipalities are given tools to help them stimulate healthy behaviour and influence circumstances to improve residents’ health, such as materials for interventions recognized to be effective and active intervention elements. The government recognizes the importance of adapting parts of existing interventions to neighborhood situations and target populations.

### Strength and limitations

Our study had several strengths. First, previous research has mainly focused on traditional health behaviours (diet, PA and smoking) and the well-being of older adults. In our study, we also included social activity as a health behaviour. which seems to be essential for the well-being for older Surinamese adults. Second, in the current study specific food items such as Surinamese vegetables (e.g. bitawiri, bravoe) were given as examples, to take the diet habits of Surinamese people into account. Third, despite the diversity in the Surinamese population in the Netherlands, we included all ethnic groups in our study. Given the heterogeneity of the Surinamese population in the Netherlands, additional analyses were performed to examine whether ethnicity (Surinamese Chinese, Surinamese Creole, Surinamese Javanese, and Surinamese Hindustani) significantly affected well-being. No significant differences in well-being were found among the ethnic groups (Additional file 1). Fourth, we collected data during the onset of the COVID-19 pandemic, which gives us valuable information about health behaviours of a vulnerable group in the Netherlands. In order to examine whether well-being levels differed between participants who filled in the questionnaire before and after COVID-19 measures, a separate analysis was performed. Reported well-being levels did not decrease after the implementation of the COVID-19 measurements (Additional file 2).

Our findings should be viewed in light of the study’s limitations. First, the study is a cross-sectional survey, which does not permit to make causal inferences. Health behaviours and well-being might be reciprocally related. For example, research indicates that older adults with a higher well-being level tend to have a healthier diet, compared to older adults with a lower well-being level [63]. Future studies should explore the effects of changes in well-being on health behaviours. Second, health risk behaviours such as not meeting the PA goal and irregular eating patterns tend to cluster together in ethnic minority groups but not in native Dutch people [64]. However, in our study we did not examine this. Future research could investigate these clusters, including social activity. among the Surinamese older population to develop prevention strategies. Third, our response rate was relatively low, which might indicate response bias. We conducted non-response analyses, which revealed significant differences in age and gender between responders and non-responders. There were more females among non-respondents than respondents (60.3% vs. 53.8%), but the effect size was small (phi = –0.058, *p* = 0.003). Respondents were slighter younger than non-respondents (mean age, 76.23 [SD = 4.93] vs. 76.80 [SD = 5.46] years, respectively; Cohen’s d = 0.106, *p* < 0.001). This difference might indicate selective non-response, but we expected a low response rate as the involvement of older non-Western migrants, such as older Surinamese adults, in research is known to be challenging [65, 66]. Moreover, some older adults may not have been able to complete the questionnaire because they were too vulnerable (e.g. health), which may have resulted in the overestimation of the well-being level in the total population. However, this possibility did not influence our main conclusions, as we focused on the relationships between health behaviours and well-being. Fourth, the use of self-administered questionnaires alone to measure PA is a limitation of this study. We collected the study data during the COVID-19 pandemic, which made home visits problematic or impossible. Thus, we did not use objective measures of physical activity such as walking or fitness tests. Fifth, we did not include items covering all potentially relevant aspects in the questionnaire. For example, we did not ask participants about their oral health, which is known to affect dietary behaviour and to be essential for good health and well-being [67–70]. We also did not assess participants’ acculturation, length of residence in the Netherlands, age at migration, cognition, or independence in (instrumental) activities of daily living, which may be associated with well-being or health [71–76]. Future research on health behaviours and well-being should involve the consideration of these aspects.

## Conclusion

From this study, we can conclude that multiple health behaviours are associated with better well-being among the older Surinamese population. Next to traditional health behaviours (healthy diet and physical activity), social activity (being able to visit others on a regular basis) is associated with the well-being of older adults with a Surinamese background. Since, social participation is still an undervalued health behaviour, intervention designers should involve this. These findings represent a first step toward developing health behavior interventions and policies to improve the well-being of older Surinamese adults. Policy makers designing health promotion strategies should aim to enhance healthy dietary habits and physical and social activity among older Surinamese adults in the Netherlands to promote their well-being.

## Supplementary Information

**Additional file 1. **Well-being Before and After Implementation of COVID-19 Measures*.

**Additional file 2. **Well-being of Surinamese Creole, Surinamese Hindustani and Other Surinamese*.

## Data Availability

The dataset analyzed during the current study is available from the corresponding author on reasonable request.

## Abbreviations

ANBO: Algemene nederlandse bond voor ouderen (General Dutch Association for Older Adults)
COVID- 19: Corona virus disease 2019
PA: Physical activity
SPF-IL: Social production function instrument for the level of well-being
STROBE: Strengthening the reporting of observational studies in epidemiology
