# Supplementary material for: Health behaviours and well-being among older adults with a Surinamese migration background in the Netherlands
Source: BMC Public Health. 2022 Nov 2;22:2006. doi: 10.1186/s12889-022-14414-z (PMC9628019; doi:10.1186/s12889-022-14414-z)
Supplement: Supplementary file 1 — Additional file 1. Well-being Before and After Implementation of COVID-19 Measures*. [file 12889_2022_14414_MOESM1_ESM.docx]

**Additional file 1. Well-being Before and After Implementation of COVID-19 Measures***

|  | Before COVID-19 measures  (*n* = 200) | After COVID-19 measures  (*n* = 422) | *p* |
| --- | --- | --- | --- |
|  | Mean (SD) | Mean (SD) |  |
| Well-being | 2.9 (.47) | 2.9 (4.7) | 1.0 |

*One way paired t-test was performed in order to compare well-being among participants who filled in the questionnaire before and after the implementation of COVID-19 measures in the Netherlands.

SD, standard deviation.
