# Supplementary material for: Health behaviours and well-being among older adults with a Surinamese migration background in the Netherlands
Source: BMC Public Health. 2022 Nov 2;22:2006. doi: 10.1186/s12889-022-14414-z (PMC9628019; doi:10.1186/s12889-022-14414-z)
Supplement: Supplementary file 2 — Additional file 2. Well-being of Surinamese Creole, Surinamese Hindustani and Other Surinamese*. [file 12889_2022_14414_MOESM2_ESM.docx]

**Additional file 2. Well-being of Surinamese Creole, Surinamese Hindustani and Other Surinamese***

|  | Surinamese Creole (n=277) | Surinamese Hindustani (250) | Other (n=129) | *p* |
| --- | --- | --- | --- | --- |
|  | Mean (SD) | Mean (SD) | Mean (SD) |  |
| Well-being | 2.90 (.47) | 2.82 (.47) | 2.90 (.47) | .081 |

* Analysis of Variance (ANOVA) was performed in order to compare well-being among participants based on their ethnicity.
SD, standard deviation.
